# Comparison of Panoramic Radiography and Cone Beam Computed Tomography in Assessing the Position of Impacted Maxillary Canines

**DOI:** 10.7759/cureus.91508

**Published:** 2025-09-02

**Authors:** Ahmet E Karabiyik, Ayse P Sumer

**Affiliations:** 1 Maxillofacial Radiology, Bursa Oral and Dental Health Hospital, Bursa, TUR; 2 Faculty of Dentistry, Department of Maxillofacial Radiology, Ondokuz Mayis University, Samsun, TUR

**Keywords:** buccopalatal position, diagnostic accuracy, facial symmetry, impacted maxillary canine, malocclusion

## Abstract

Introduction: This study aimed to assess the clinical applicability of a panoramic radiograph (PR)-based threshold angle for distinguishing the buccopalatal position of impacted maxillary canines, using cone-beam computed tomography (CBCT) as a reference for verification.

Methods: A total of 152 impacted maxillary canines from 129 patients were evaluated. The angle of each impacted canine was measured on PR between the long axis of the tooth and the intermolar line. CBCT was used as a reference standard to confirm the buccopalatal position of each canine. Receiver-operating characteristic (ROC) analysis was performed to determine the optimal PR-based angle threshold value for distinguishing buccal and palatal impactions.

Results: The intraobserver reliability for PR angle measurements was excellent (intraclass correlation coefficient (ICC) = 0.957). An optimal PR angle threshold value of 50.4° was identified for differentiating buccal from palatal impactions. However, this value did not achieve statistical significance in the overall cohort (p = 0.106; sensitivity = 52.6%, specificity = 70.2%). In the subgroup of unilaterally impacted canines, the same threshold value reached statistical significance (p < 0.05) with a sensitivity of 62% and a specificity of 75%. Despite these findings, the predictive accuracy of PR remained insufficient for reliable clinical application.

Conclusion: The study demonstrated that a PR-based angle threshold value of 50.4° can provide a preliminary estimation of the buccopalatal position of impacted maxillary canines, but its diagnostic accuracy is limited. CBCT remains the preferred method for definitive localization.

## Introduction

Impacted maxillary permanent canines are among the most common tooth position anomalies, influenced by various etiological factors. These impactions can occur unilaterally or bilaterally and may disrupt the symmetry of the dental arch in all three spatial dimensions: sagittal, vertical, and transverse [1].

Several factors contribute to canine impaction, including a long eruption pathway, their position as the last teeth to erupt in the anterior maxilla, and their deeper developmental location. Additionally, pathological conditions, malocclusion, ectopic positioning, persistence of deciduous teeth, the presence of supernumerary teeth, and soft or hard tissue obstructions can prevent eruption [2].

Early detection, timely intervention, and appropriate orthodontic and surgical treatment are critical for the proper alignment of impacted canines within the dental arch. Imaging techniques play a crucial role in evaluating the position of impacted teeth and formulating an appropriate treatment plan. Traditional two-dimensional (2D) radiographic techniques, including panoramic radiographs (PR), occlusal radiographs, and periapical radiographs, remain the most widely used methods for the initial diagnosis, localization, and treatment planning of unerupted canines [3].

PRs are frequently employed to assess the inclination of maxillary canines, which is considered a key indicator of the risk of impaction and its associated complications. Several methods have been proposed in the literature to measure canine inclination, and these techniques have demonstrated high reproducibility [4].

Cone-beam computed tomography (CBCT) provides highly accurate images for determining the position and angulation of impacted canines in the buccopalatal dimension. It is particularly useful for assessing the proximity of impacted canines to adjacent incisor and premolar roots, as well as the extent of root resorption [5]. These factors are essential in treatment planning to facilitate the proper movement of the impacted canine and minimize the risk of resorption in neighboring teeth.

For the comprehensive evaluation of impacted canines, 2D radiographs often prove insufficient, particularly in assessing critical factors such as buccopalatal positioning, tooth angulation, root dilacerations, and resorption in adjacent teeth. Moreover, the developmental stage of the tooth is a crucial consideration in treatment planning. Weiss et al. conducted a systematic review on the role of CBCT in dentoalveolar surgery, dental implants, temporomandibular joint (TMJ) assessment, orthognathic surgery, trauma evaluation, and pathology diagnosis, including preoperative and postoperative surgical planning. Their findings highlighted the superiority of CBCT in accurately determining tooth dimensions, angulation, and root resorption. Similarly, recent comparative studies between 2D and 3D radiographic imaging have consistently demonstrated that CBCT provides superior diagnostic accuracy in assessing tooth morphology and resorption [6].

Therefore, in cases where 2D radiographs fail to provide sufficient anatomical detail prior to the surgical management of impacted canines, CBCT is recommended. To minimize radiation exposure, the smallest possible field of view should be selected [7].

This study examines the use of two different imaging modalities in assessing the buccopalatal position of impacted maxillary canines. The spatial position was assessed based on the inclination angle measured on PR. If the inclination angle exceeded the threshold value, the tooth was considered buccally impacted, whereas if it fell below the threshold value, it was considered palatally impacted. CBCT defined buccal impaction as the crown apex of a canine tooth positioned more buccally compared to the root apex of an adjacent tooth. Conversely, a more palatal position of the crown apex was considered palatal impaction.

Using CBCT as a validation reference, the study seeks to answer the following question: Can the buccopalatal spatial position of impacted maxillary canines be reliably determined using only two-dimensional radiographic data, without resorting to three-dimensional imaging methods?

## Materials and methods

This study was reviewed by Ondokuz Mayıs University Clinical Research Ethics Committee and found ethically appropriate with the decision number 2021/145 dated 25.03.2021.

This study retrospectively analyzed images of patients who underwent PR and CBCT between January 2020 and December 2021 at the Department of Dentomaxillofacial Radiology. These imaging procedures were performed for various clinical indications, including impacted teeth, implant planning, and the evaluation of jaw lesions.

Inclusion criteria

Patients meeting the following criteria were included in the study: 1. Patients with completed craniofacial growth that has reached a stable occlusal position (Age 18 years or older), 2. Presence of unilateral or bilateral impacted maxillary canines, 3. Fully erupted adjacent maxillary teeth and maxillary molars, 4. Availability of PR and CBCT images with adequate diagnostic quality, 5. PR and CBCT images obtained simultaneously, 6. No history of dental trauma or surgical procedures in the anterior maxilla

Exclusion criteria

Patients were excluded if they met any of the following criteria: 1. Patients with incomplete craniofacial growth that has not yet reached a stable occlusal position (Age under 18 years), 2. Completely edentulous patients, 3. Impacted canines in an inverted or horizontal position, 4. Fully erupted maxillary canines, 5. PR and CBCT images of inadequate diagnostic quality, 6. Absence of PR and CBCT images taken at the same time

PR and CBCT imaging system

The PR images were obtained with a Sirona Ortophos XG3 (Sirona Dental Systems, Bensheim, Germany) device operating at a maximum of 90 kVp and 12 mA with an irradiation time of 14 s. CBCT images were obtained with a dental volumetric imaging system (GALILEOS Comfort Plus, Sirona Dental Systems, Bensheim, Germany) operating at 98 kVp and 15-30 mAs. CBCT images were created with 15 mm × 15 mm FOV, 12-bit gray level, 0.25 mm isotropic voxels, 14 s exposure time, 612-1139 mGy × cm2. Simultaneous reconstruction was performed with Sirona Sidexis XG 2.61 imaging software. All examinations were performed on a 3.7 MP, 68 cm, 2560 × 1440 resolution, 27-inch color LCD screen (The RadiForce MX270W, Eizo Nanao Corporation, Ishikawa, Japan).

Evaluation of images

Analysis of PR and CBCT images was conducted by an experienced dentomaxillofacial radiologist under dim lighting conditions. To assess intraobserver reliability, 50 PR images were re-evaluated by the same observer for measurements, with a three-week interval between the two assessments. The intraobserver agreement was analyzed based on the obtained data.

The sample size of the study was determined based on an a priori power analysis. Using a significance level of α = 0.05 and a statistical power of 80%, the minimum required sample size was calculated. The inclusion of 152 impacted teeth in the present study met this requirement.

A total of 152 impacted maxillary canines from 129 patients who met the inclusion criteria were included in the study. The distribution of impacted canines was evaluated in 44 male and 85 female patients using both PR and CBCT images.

**Figure 1:**
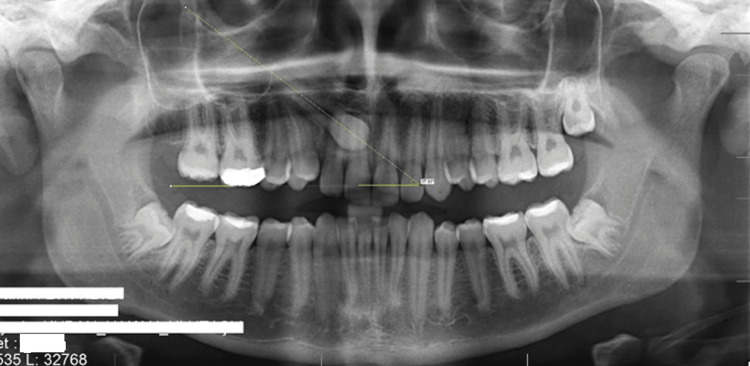
Angle measurement on panoramic radiograph. The angle between the two yellow lines passing through the mesiobuccal cusp of the first molar tooth and the long axis of the impacted canine tooth was recorded.

The study variables included age, gender, and the position of impacted canines. In PR, the inclination angle of the impacted maxillary canine was measured. This angle was determined by the intersection of two reference lines: (1) A horizontal line was drawn from the mesiobuccal cusp tip of both the right and left maxillary first molars, and (2) along the long axis of the impacted canines (Figure 1).

**Figure 2:**
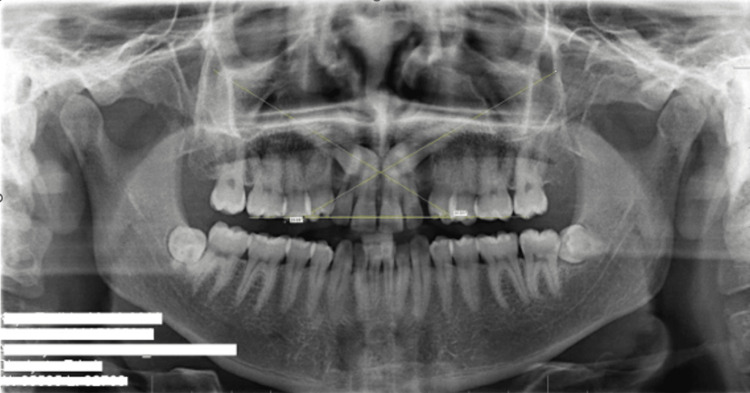
The angle measurement applied to both sides of bilaterally impacted teeth.

The inclination of the canine, measured laterally to the midline, was recorded in degrees. For unilateral impactions, a single angle was recorded, whereas for bilateral impactions, the angulation of both canines was documented (Figure 2).

The localization of impacted maxillary canines was determined using CBCT by evaluating the relationship between the canine crown and the roots of adjacent teeth in the buccopalatal dimension. If the crown of the impacted canine was positioned buccally relative to the adjacent tooth root, it was classified as a buccally localized impaction; if positioned palatally, it was classified as a palatally localized impaction.

To determine the precise localization of impacted maxillary canines, sagittal, coronal, axial, cross-sectional, and 3D reconstructions were analyzed from CBCT images.

**Figure 3:**
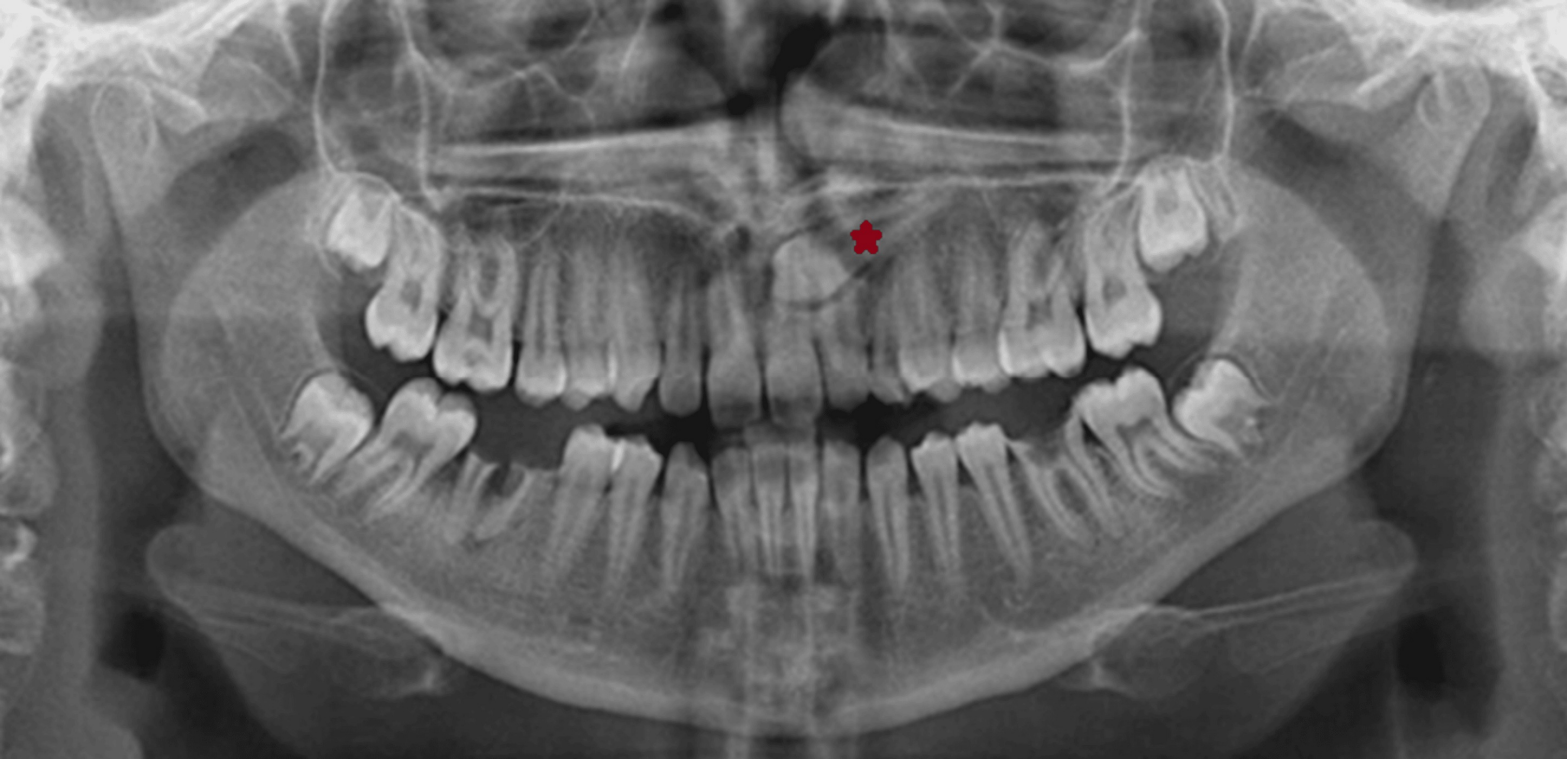
The red star on the PR shows the canine tooth positioned palatally. PR: panoramic radiograph

**Figure 4:**
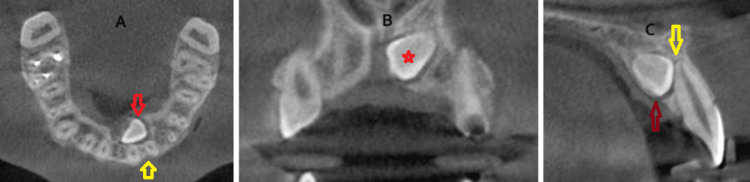
CBCT view of a palatally localized impacted maxillary canine tooth. A) Axial view: red arrow shows the crown position of the impacted canine tooth; yellow arrow shows the roots of the adjacent teeth. B) Coronal view: red star shows the coronal position of the impacted canine tooth. C) Sagittal view: red arrow shows the crown apex position of the impacted canine tooth; yellow arrow shows the root apex of the adjacent tooth. CBCT: cone-beam computed tomography

Representative 2D and 3D images of a palatally localized impacted maxillary canine are shown below (Figures 3-4A-4C).

**Figure 5:**
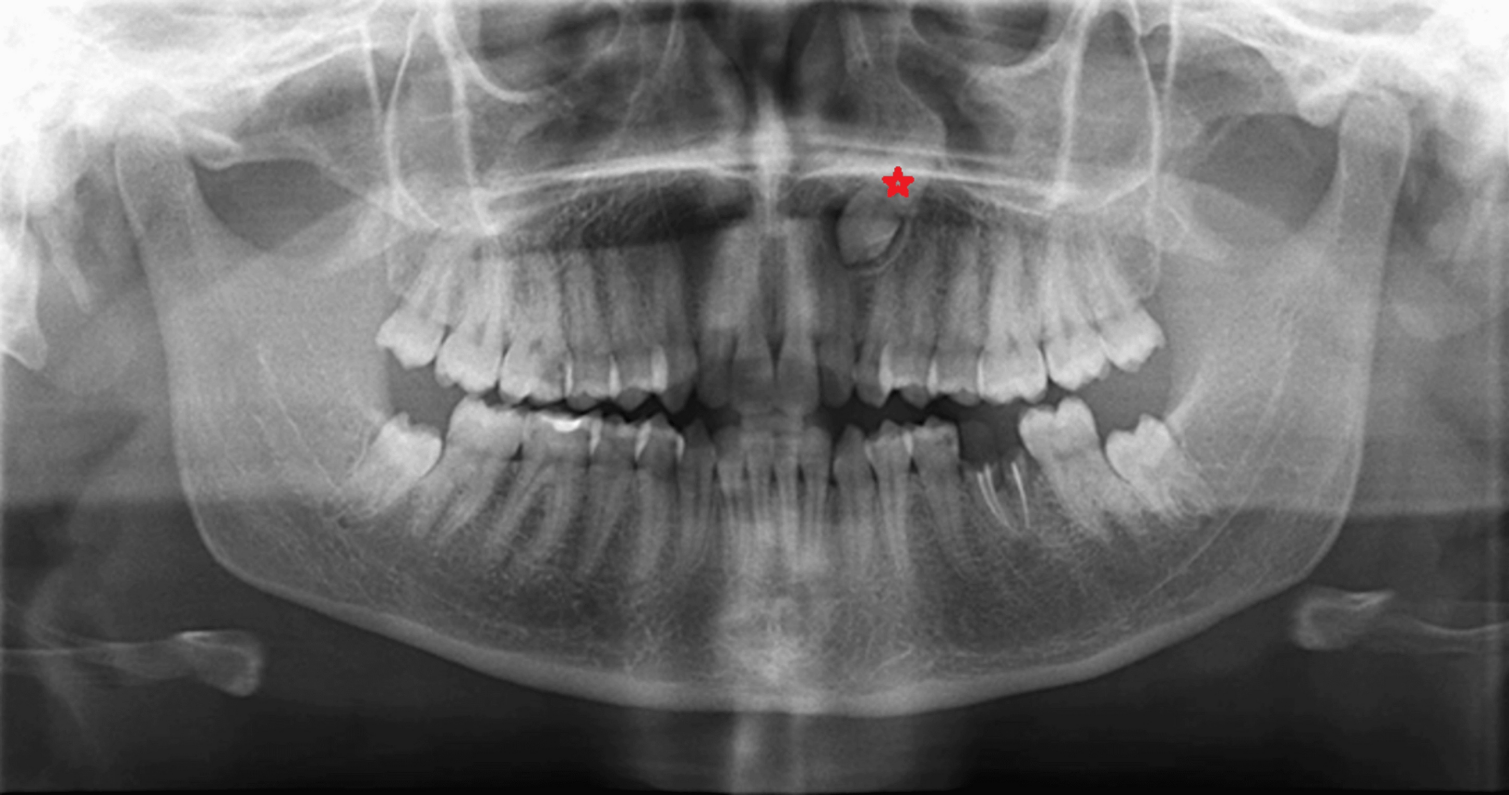
The red star on the PR shows the canine tooth positioned buccally. PR: panoramic radiographs

**Figure 6:**
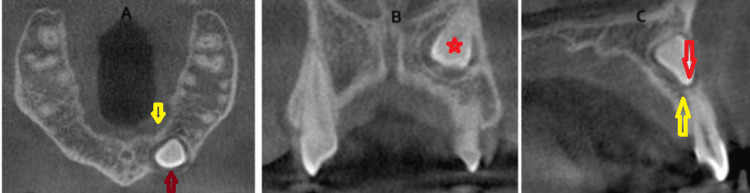
CBCT view of a buccally localized impacted maxillary canine tooth. A) Axial view: red arrow shows the crown position of the impacted canine tooth; yellow arrow shows the palatal alveolar bone. B) Coronal view: red star shows the coronal position of the impacted canine tooth. C) Sagittal view: red arrow shows the crown apex position of the impacted canine tooth; yellow arrow shows the root apex of the adjacent tooth. CBCT: cone-beam computed tomography

Representative 2D and 3D images of a buccally localized impacted maxillary canine are shown below (Figures 5-6A-6C).

Statistical analysis

Statistical analyses were performed using SPSS software, version 15.0 (Chicago, Illinois, USA). Because age values did not conform to a normal distribution, the Mann-Whitney U test was used for pairwise comparisons. Before conducting statistical tests, the Shapiro-Wilk test was applied to assess the normality of continuous variables.

For normally distributed data, the Student’s t-test was applied, whereas for non-normally distributed data, the Mann-Whitney U test was used. The Chi-square test was employed for the comparison of categorical variables.

To assess intraobserver reliability for PR angle measurements, intraclass correlation coefficients (ICC) were estimated based on repeated evaluations conducted by the same observer. A p-value < 0.05 was considered statistically significant.

## Results

Among the 129 patients included in the study, 44 (34.1%) were male and 85 (65.9%) were female. The mean age of all patients was 33.8 ± 14.2 years (range = 18-75 years). The mean age of male patients was 33.9 ± 13.1 years (range = 18-60 years), while the mean age of female patients was 33.7 ± 14.8 years (range = 18-75 years). There was no statistically significant difference in age between males and females (p = 0.915).

The intraobserver agreement for PR angle measurements was found to be high (ICC = 0.957), indicating good reliability.

The mean PR angle for all patients was 44.1°. The mean PR angle for males was 45.6° ± 18.5° (range = 14.6°-89.6°), while the mean PR angle for females was 43.5° ± 19.9° (range = 6.5°-88°). The difference between male and female patients was not statistically significant (p = 0.523) (Table 1).

**Table 1:** The mean age and PR angle of impacted canine teeth, stratified by gender, are presented in the table. PR: panoramic radiograph

	n	Mean Age	Mean PR Angle (°)
Gender			
Male	44	33.9	45.6
Female	85	33.7	43.5
Total	129	33.8	44.1

In the entire study group, 38 (25.0%) of the impacted maxillary canines were buccally localized, while 114 (75.0%) were palatally localized. Regarding the side distribution, 81 (53.3%) of the impacted canines were located on the right side, whereas 71 (46.7%) were on the left side (Table 2).

**Table 2:** The prevalence of impacted canines by gender and location, as assessed on panoramic radiographs. PR: panoramic radiograph

	Buccal (%)	Palatal (%)	p-value
Gender			1.000
Male	12 (31.6)	36 (31.6)	
Female	26 (68.4)	78 (68.4)	
PR localization			0.709
Right	19 (50.0)	62 (54.4)	
Left	19 (50.0)	52 (45.6)	
Total	38 (100)	114 (100)	

Among the patients whose radiographs were analyzed, 23 (17.8%) had bilaterally impacted maxillary canines, while 106 (82.2%) had unilaterally impacted canines.

There was no statistically significant relationship between the presence of bilaterally impacted maxillary canines and gender, although the p-value was close to statistical significance (p = 0.062). Among male patients, 9.1% had bilateral impactions, whereas among female patients, 22.4% had bilateral impactions.

Among the 106 patients with unilaterally impacted canines, 40 (37.7%) were male, and 66 (62.3%) were female. Of these unilateral impactions, 77 (72.6%) were palatally localized, while 29 (27.4%) were buccally localized. In terms of side distribution, 48 (45.3%) of the unilateral impactions were on the left side, while 58 (54.7%) were on the right side.

Among the bilaterally impacted canines, 9 (19.6%) were buccally localized, whereas 37 (80.4%) were palatally localized. In 19 patients, both impacted canines were on the same side (buccal-buccal: n=2; palatal-palatal: n=17), while in 4 patients, the impactions were on opposite sides (buccal-palatal).

The mean age of patients with buccally impacted canines was 29.9 ± 14.8 years, while the mean age of those with palatally impacted canines was 34.4 ± 14.1 years. Although this difference was not statistically significant, the p-value was close to the threshold for significance (p = 0.055).

The mean PR angle for buccally impacted canines was 48.5° ± 22.3°, while for palatally impacted canines, it was 42.7° ± 18.3°. There was no statistically significant difference in PR angles based on the impaction location (p = 0.149) (Table 3).

**Table 3:** The localization of bilaterally impacted canine teeth, the mean PR angle, and the mean age.

	n	Mean Age	Mean PR Angle (°)
Localization			
Buccal	9	29.9	48.5
Palatal	37	34.4	42.7

No statistically significant correlation was found between PR angles and age (p = 0.208).

ROC analysis evaluated the diagnostic performance of the PR angle in predicting the buccal location of impacted maxillary canines. Different angle threshold values were tested, and sensitivity and specificity were calculated for each threshold point. The optimal threshold value was determined using the Youden Index (J = sensitivity + specificity - 1). The analysis yielded the highest Youden Index at an angle of 50.4°. Accordingly, PR angle values of 50.4° and above were considered the most appropriate threshold value for predicting the buccal location of impacted maxillary canines.

**Figure 7:**
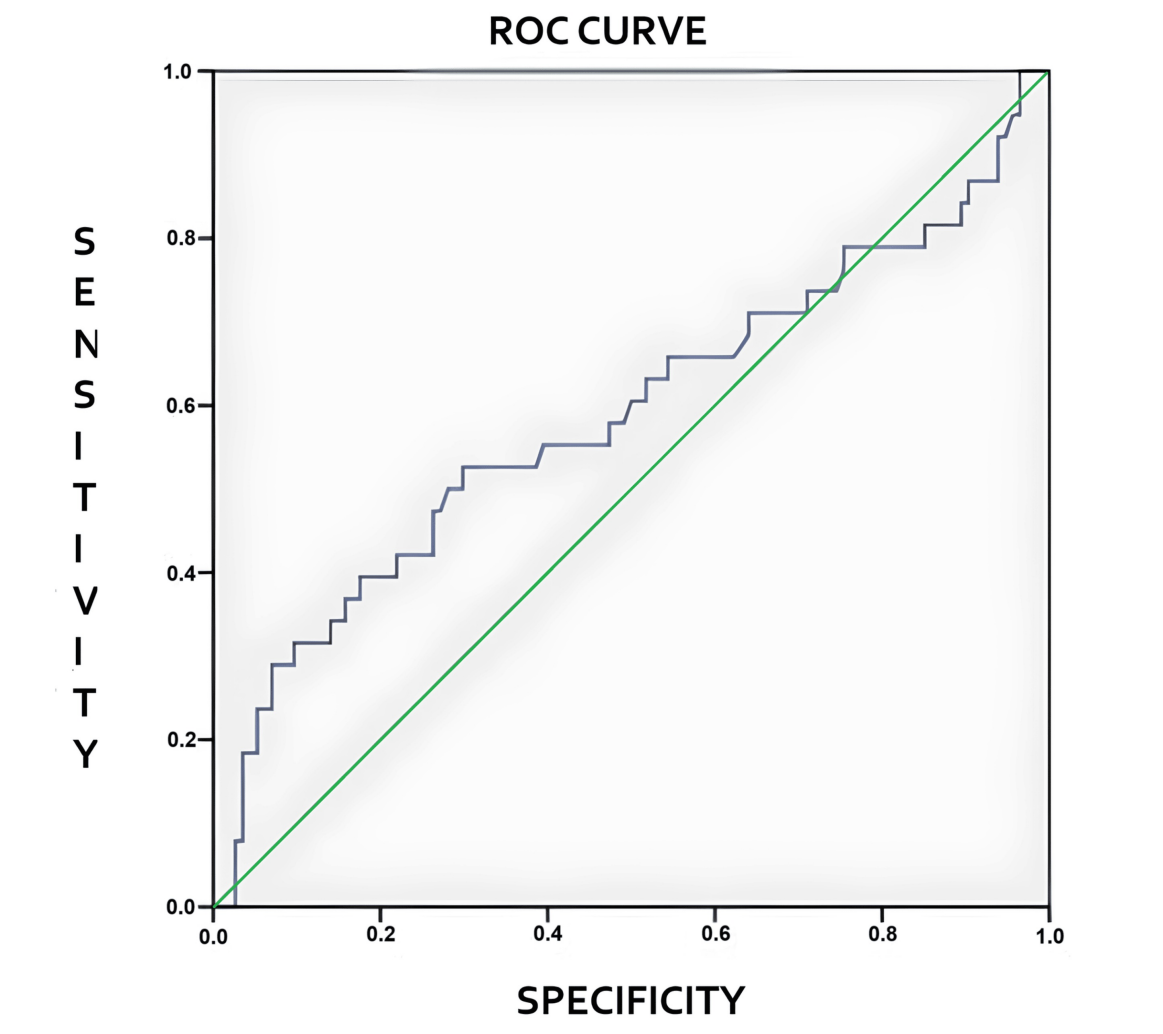
The ROC curve is presented in the figure. Green diagonal line. If the curve is close to this line, the model is indistinguishable from random guessing. Blue line: Classification performance obtained from panoramic radiograph angles. ROC: Receiver-operating characteristic

When all impacted canines were analyzed, the optimal threshold value for distinguishing between buccal and palatal localization was found to be 50.4° (area under the curve (AUC) = 0.588, 95% confidence interval (CI) = 0.471-0.705). However, this result was not statistically significant (p = 0.106). For this threshold value, the sensitivity was 52.6%, and the specificity was 70.2% (Figure 7).

When only unilaterally impacted canines were analyzed and ROC analysis was performed, the optimal threshold value for distinguishing between buccal and palatal localization was found to be 50.4°. This value was statistically significant (AUC = 0.661, 95% CI = 0.530-0.793, p = 0.011). When the threshold value of 50.4° was applied, the sensitivity was 62%, and the specificity was 75%.

In both unilateral and bilateral impaction analyses, and in the unilateral impaction-only analysis, the best threshold value was consistently determined as 50.4°. However, due to insufficient specificity and sensitivity, it was concluded that a 50.4° angle cannot reliably predict the buccal or palatal localization of impacted maxillary canines.

## Discussion

Maxillary canines play a crucial role in facial aesthetics, dental arch development, and occlusion. Generally, a significant inclination, an increased distance from the occlusal plane, or mesial displacement is associated with a higher risk of canine impaction. The buccal or palatal localization of impacted canines, along with their inclination, is essential for treatment planning and determining the most effective therapeutic approach.

Various methods have been proposed to determine the position of impacted maxillary canines, including clinical examination, Clark’s method, magnification assessment, computed tomography (CT), and CBCT [8]. Among these, 3D imaging techniques such as CBCT and CT are considered the gold standard for evaluating anatomical structures, pathologies, and impacted teeth. However, these techniques are not always accessible, costly, and expose patients to higher radiation doses than conventional imaging methods. Consequently, studies have focused on estimating the localization of impacted canines using PRs, which are routinely obtained during standard dental examinations. The findings from such studies are particularly relevant in assessing whether a single PR can provide sufficient information for initial diagnosis, potentially reducing the need for advanced imaging techniques [9,10,11].

A study by Ismail et al. evaluated the potential of PR in predicting the risk of impacted maxillary canines in 9- to 10-year-old children [12]. Their results indicated that approximately 85% of canine positions fell within an acceptable confidence interval, demonstrating that PR was effective for predicting tooth localization, guiding treatment planning, and preventing possible complications. Based on their findings, they concluded that PR is a valuable diagnostic tool in the early assessment of impacted canines.

Similarly, in a systematic review by Alamri et al., 16 studies were selected from a total of 221 articles to evaluate canine position assessment methods [13]. Among these, studies relied solely on PR, three studies used both PR and CBCT, and only one study relied exclusively on CBCT [12]. Their analysis concluded that maxillary canines were more frequently impacted, with a higher prevalence in females, on the left side, and predominantly in a palatal position. The findings of our study align with those of Alamri et al., as we observed a higher prevalence of impacted maxillary canines in females (65.9%) and a predominance of palatal impactions (75%) [13]. However, in contrast to their study, we found that impacted maxillary canines were more frequently located on the right side in our study population. Our results are also consistent with the findings of Grisar et al., who reported that impacted maxillary canines are more frequently observed in females and are predominantly unilateral [14].

Various methods have been employed in the literature to assess the localization of the canine tooth and the risk of impaction. One such method involves measuring the external angle formed by the long axis of the canine tooth and a straight line passing through both suborbital points. Another approach considers the internal angle formed between the long axis of the canine tooth and the “bicondylar line”, which connects the uppermost points of the right and left condyles [15,16]. Additionally, Ericson and Kurol introduced a sectoral method based on the position of the cusp tip of the impacted canine [17]. In 2011, Sigler et al. proposed the Alpha angle (the mesial slope of the permanent canine crown relative to the midline), the D distance (the distance from the cusp tip of the permanent canine to the occlusal line), and the Sector classification (the mesial position of the displaced canine crown in relation to the central and lateral incisors) [18]. In 2019, Alejos-Montante et al. employed a sectoral method modified by Lindauer et al. and the Power and Short (PS) geometric measurement analysis [9,19]. In the PS method, a perpendicular line was established as the midline, along with a horizontal reference plane. A mesial angular measurement was obtained from the midline to the longitudinal axis of the impacted canine. According to this method, angles between 0° and 30° were classified as low risk for canine impaction, while angles exceeding 31° were considered indicative of a high risk of impaction [20].

Katsnelson et al. analyzed patients with at least one impacted maxillary canine and evaluated anatomical measurements along with the angulation of the impacted canines in PR [21]. In their study, a horizontal reference line was drawn from the mesiobuccal tubercles of the right and left maxillary first molars, and a second line was drawn along the long axis of the impacted canine. The inclination angle of the impacted canine relative to the horizontal reference line was recorded in degrees. Their analysis included 130 impacted maxillary canines from 102 patients, of which 59 were buccally localized and 71 were palatally localized. The angulation of the impacted maxillary canines relative to the horizontal reference line passing through the mesiobuccal tubercles of the maxillary molars was measured, and these measurements were used to estimate the position of the impacted canines and correlate these estimations with the actual surgical findings. The study reported that the mean inclination angle for buccally localized impacted maxillary canines was 75.1° ± 18.2° (range = 8°-111°), whereas the mean inclination angle for palatally localized impacted maxillary canines was 51.3° ± 15.3° (range = 12°-91°). Notably, it was found that impacted maxillary canines with an angulation greater than 65° were 26.6 times more likely to be localized on the buccal side than on the palatal side. In the present study, the position of impacted maxillary canines was evaluated using the same measurement technique as described by Katsnelson et al., with a modified sample group. The aim here was to test the clinical applicability of this technique using a more reliable imaging method. However, the threshold angle value changed when the sample group changed. In this study, the threshold angle was found to be 50.4°. However, this threshold value did not provide sufficient reliability. The difficulty in producing images in a standardized manner, due to distortion and magnification in two-dimensional imaging, as well as the difficulty in reproducing reference points in the applied techniques, may be the reason for the unreliability of the results. It was concluded that an assessment using this method could only provide the clinician with a prediction, but was not a reliable method.

An et al. analyzed 102 impacted maxillary canines from 94 patients, evaluating magnification, angulation, and superposition on PR [22]. The exact localization of the impacted teeth was determined using CBCT sections. The angulation method used in their study was based on the technique described by Katsnelson et al., which was also applied in our study [21]. This method is founded on the premise that an impacted maxillary canine with an angulation greater than 65° to the horizontal plane is likely to be positioned buccally. Their findings indicated that 49 of the impacted canines were buccally localized, while 53 were palatally localized. They reported that magnification and angulation methods based on a single PR were not reliable for determining the position of impacted canines. However, superposition analysis, when combined with other assessment methods, was found to be helpful for localization. In our study, similar to the findings of An et al., PR-based angle measurements were not effective in accurately determining the buccal or palatal position of impacted maxillary canines [22].

Ismail et al. obtained successful results in a study conducted on a younger population [12]. In contrast, An et al. failed to achieve reliable outcomes when applying similar techniques to an older patient group. In the present study, individuals in an even more advanced age group - where jaw development and tooth eruption were already complete - were examined, and similarly, unreliable results were obtained. These findings, as emphasized by Ismail et al., indicate that techniques applied to two-dimensional radiographs can be useful in detecting impacted teeth and predicting early preventive treatments in younger populations. However, in older age groups - particularly in cases requiring surgical intervention and more detailed evaluation of the region - CBCT should be considered the preferred imaging modality. This conclusion is also consistent with the review conducted by Alamri et al. [13].

Recent studies have explored the use of artificial intelligence (AI) models for evaluating the localization of impacted maxillary canines. Swaity et al. utilized a convolutional neural network (CNN) model for the automatic segmentation of impacted maxillary canines on CBCT images [23]. Their results demonstrated that the model was capable of performing segmentation quickly, consistently, and accurately. Minhas et al. applied a deep learning reconstruction algorithm trained on PR and pseudo-3D images of 123 patients created using the Sandbox module [24]. The algorithm predicted the buccal, medial, or palatal position of impacted canines with 41% accuracy, while the mesial and distal positions were predicted with 55% accuracy. Pirayesh et al. used deep learning techniques to automatically detect the presence of root resorption in maxillary incisors caused by impacted canines [25]. Their study utilized 50 CBCT images to train the model.

These studies suggest that deep learning algorithms hold promising potential for applications in dentistry. However, further development and refinement are needed to enhance the accuracy of AI-based reconstruction models for clinical use.

## Conclusions

Impacted maxillary canines are more commonly observed in females, unilaterally, and in a palatal position. Angle measurements obtained from PR were found to be unreliable for determining the buccopalatal localization of impacted canines. While PR is useful in the initial assessment of impacted maxillary canines, it does not provide sufficient accuracy for exact localization. CBCT remains the most reliable imaging modality for precise localization and treatment planning. By selecting appropriate diagnostic methods at the right time, radiation exposure can be minimized. A single CBCT scan, when necessary, is more effective and diagnostically valuable than obtaining multiple conventional radiographs. The retrospective design of the study, the fact that it was conducted at a single center, and that all measurements were performed by a single observer are among the limitations of the research. In future studies, the preference for prospective and multicenter designs, the comparison of measurements performed by different observers, and the support of analyses with larger sample groups will improve the results.
